# Study of Positioning Accuracy Parameters in Selected Configurations of a Modular Industrial Robot—Part 1

**DOI:** 10.3390/s25010108

**Published:** 2024-12-27

**Authors:** Marcin Suszyński, Marcin Wiśniewski, Kajetan Wojciechowicz, Marek Trączyński, Marcin Butlewski, Vit Cernohlavek, Rafał Talar

**Affiliations:** 1 Institute of Mechanical Technology, Poznan University of Technology, 60-965 Poznan, Poland; 2 Faculty of Mechanical Engineering, University of Jan Evangelista Purkyne in Ustí nad Labem, 40096 Ustí nad Labem, Czech Republic

**Keywords:** industrial robot, modular robot, robot accuracy, robot configuration

## Abstract

This article presents the fundamental principles of robot accuracy. It characterizes a modular robot, describes the measurement setup, and outlines the methodology for evaluating positioning accuracy across different configurations of the modular robot (four, five, and six modules) under varying loads of 6, 10, and 16 kg. An analysis was conducted on the impact of load changes on four- and five-module configurations, as well as the effect of configuration changes on the robot’s performance with 6 and 10 kg loads. The findings indicate that both the number of modules and the load affect positioning accuracy. This article highlights the importance of selecting the optimal configuration based on planned industrial tasks to ensure the highest precision and operational efficiency.

## 1. Introduction

Recently, modular robots have been gaining popularity in the global industrial automation market. Von Neumann first introduced the concept of modular robots in his “Theory of Self-Reproducing Cellular Automata” in the 1960s [1], which involved assembling homogeneous components to form systems. In 1988, the first modular cellular robot (CEBOT) was developed, comprising various types of modules, such as rotary joints, telescopic arms, and gripping units [2]. Modular industrial robots offer user-friendly, software-based automation solutions for industrial production. The modular robotics system provides complete freedom in configuring robots and cobots. Scalable and easily connectable motor modules and links enable the creation of custom robotic solutions that can be expanded and modified at any time. Such solutions are, in a sense, subsequent stages of the development of classic industrial robots, alongside the increasingly used, developed and researched collaborative robots [3,4,5].

In recent years, several studies have explored various aspects of modular robotics, including their adaptability, scalability, and potential for industrial applications. Foundational works, such as Yang and Chen’s Modular Robots: Theory and Practice [6] and the Handbook of Robotics [7], have discussed the principles of modular robot design, focusing on the benefits of reconfigurable systems. A more recent publication [8] carried out comprehensive research covering the entire assumptions and construction of modular reconfigurable robots, from their creation in 1985 to 2023. In [9], the authors focus on studying the modular robot’s configuration design and self-reconfiguration process and propose an optimized ant colony algorithm for reconfiguration path planning and verifying superiority and rationality. However, these mentioned studies often lack detailed experimental validation, particularly under varying configurations and load conditions.

Extensive research has also been conducted on the relationship between positioning accuracy and robot design parameters. For instance, studies on improving robot accuracy with an optical tracking system are presented in [10]. In [11], the authors, on the other hand, propose combining a genetic algorithm with a lexicographic evaluation of solution candidates to optimize modular robot composition. Despite these advances, most existing research focuses on traditional robotic systems rather than modular configurations, leaving a significant gap in understanding the unique challenges posed by modularity. These works provide valuable insights but are constrained by the narrow scope of configurations or lack of systematic evaluation across diverse industrial scenarios. Moreover, there is little discussion on how these findings can guide practical applications in dynamic industrial environments.

Modular robotics thus provides enhanced flexibility, adaptability, and scalability in production processes. The modules of these robots are typically standardized and can be easily replaced, allowing for the construction of various robot configurations to meet specific production requirements. The core idea behind modular robots is their ability to quickly adapt and reconfigure, enabling efficient adjustments to changing production needs. Their control software is also designed to be flexible and adaptive, allowing for robots to be programmed for a wide range of tasks [12].

The modules of modular industrial robots are designed as standalone units with specific functions. These modules can include robotic arms, grippers, sensors, effectors, and other specialized components, depending on the intended application. Each module is equipped with its own computational power, communication capabilities, and power supply, enabling it to operate independently or in conjunction with other elements [13,14,15].

Communication between modules is a critical aspect of modular industrial robots. The modules are interconnected (e.g., via standard communication interfaces such as ethernet or fieldbus systems), allowing for smooth data exchange and coordination. This connectivity facilitates synchronized control and cooperation among multiple modules, leading to efficient and coordinated operations. Software, in turn, aids in the integration and coordination of individual modules and overall robot system management. Key software components for modular industrial robots include the following:

1. An operating system that serves as the foundation for managing the robot’s hardware and software components. It provides real-time control, task scheduling, and resource allocation capabilities [15]. Modular industrial robots often utilize specialized operating systems designed for real-time control, ensuring precise and timely task execution [16].
2. Middleware, which acts as an intermediary layer between the operating system and the robot’s application software [13]. It facilitates communication and data exchange between different modules and supports the integration of additional software tools, such as vision systems or machine learning algorithms [14,17]. Middleware plays a crucial role in achieving interoperability and modularity within the robot system.
3. An appropriate development environment. It is essential for creating and modifying robot tasks. Modular industrial robots provide programming interfaces that allow for users to develop and customize applications based on their specific production needs. These development environments often include graphical user interfaces (GUIs), high-level programming languages, and libraries for robot control and motion planning [2].

Despite the numerous advantages of modular robots, maintaining positioning accuracy under changing configurations and load conditions remains a key challenge. Positioning accuracy is critical in many industrial applications, such as assembly, welding, and material handling, where even small deviations can reduce efficiency, safety, and product quality. However, the relationship between the number of modules, payload, and resulting positioning accuracy remains relatively underexplored.

The goal of this study is to analyze how modular configurations and varying loads influence the positioning accuracy of industrial robots. Specifically, this work seeks to address the following research questions:

- How does the number of modules affect a robot’s ability to achieve precise and repeatable positioning within its workspace?
- How do varying loads impact the positioning accuracy of modular robots in different configurations?
- How can insights from these relationships be utilized in practical applications to optimize the performance of modular robots in real-world industrial conditions?

This study aims to address these gaps by investigating the relationship between modular configurations, load conditions, and positioning accuracy in a systematic and experimentally validated manner. By building on prior research and focusing on four-, five-, and six-module configurations, this work seeks to provide actionable guidelines for optimizing modular robots for real-world industrial tasks.

The selected configurations represent a balance between real-world industrial requirements and experimental constraints. The analysis of the results provides actionable guidelines for optimizing modular robots in various industrial environments.

## 2. Materials and Methods

### 2.1. Evaluation of Robot Positioning Accuracy

Robot positioning accuracy is a critical factor influencing its performance and suitability for various industrial applications. The number of axes in a robotic system significantly affects positioning accuracy. The relationship between the number of axes and the achievable positioning accuracy is crucial, providing essential information for selecting the robot, configuring it, and designing applications. Robots are classified based on their configuration, commonly defined by the number and arrangement of their axes. The number of axes or degrees of freedom (DOFs) determines the robot’s ability to move and position itself in three-dimensional space [18].

Positioning accuracy is quantified using various indicators to assess the robot’s ability to achieve precise and repeatable positions. Two commonly used indicators are repeatability and positioning accuracy [19,20].

**Figure 1:**
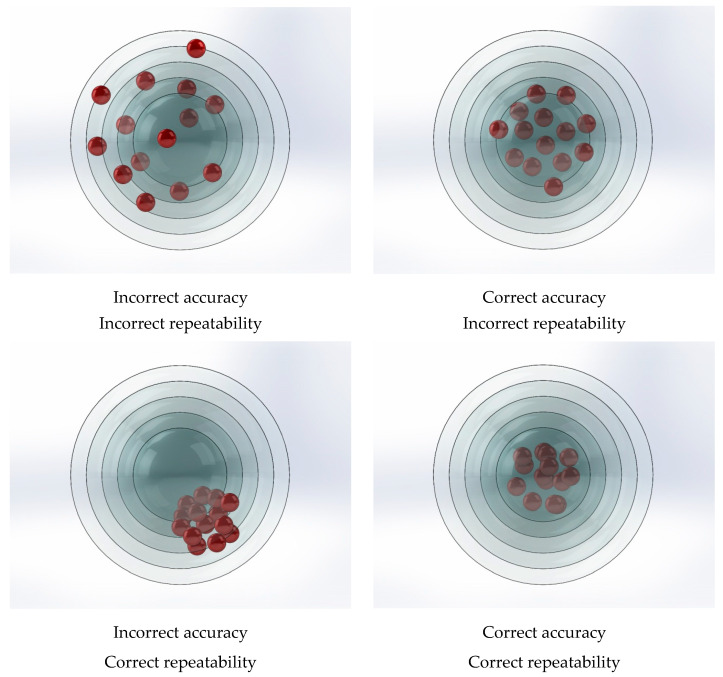
Repeatability and positioning accuracy.

Repeatability measures the robot’s ability to return to a specified position after multiple commands (Figure 1) [21]. It reflects the robot’s capacity to achieve consistent results under the same operating conditions. Repeatability is typically expressed as the distance or deviation from the target position and is influenced by factors such as mechanical backlash, control system dynamics, and sensor noise [19,20].

Accuracy, on the other hand, refers to the difference between the robot’s actual position and its intended target position. It represents systematic error or deviation in the robot’s positioning [21]. Accuracy is affected by various factors, including mechanical compliance, calibration errors, sensor precision, and kinematic inaccuracies. Accuracy is usually expressed as a percentage or an absolute distance error [19].

The number of axes in a robot system obviously impacts its positioning accuracy and repeatability. Generally, an increase in the number of axes provides greater flexibility and enables more complex movements. However, this increased flexibility can introduce additional sources of error and reduce overall positioning accuracy [22]. The relationship between the number of axes and positioning accuracy depends on various factors, including the robot’s configuration, mechanical design, control system, and calibration techniques.

### 2.2. Characteristics of the Examined Modular Industrial Robot

The modular industrial robot used in this study offers a range of features that contribute to its versatility and efficiency in industrial applications. The robot has a payload capacity of up to 20 kg (Table 1), enabling it to handle and transport heavy objects. This makes it suitable for a wide range of industrial tasks, including material handling, assembly, and packaging [21].

The robot can be programmed by manually moving its individual modules. However, it is not classified as a cobot due to the lack of appropriate certification. It is designed for seamless integration with existing automation systems and production lines. The robot supports various communication protocols and interfaces (Table 2), facilitating interoperability and integration in industrial environments [21].

It has been designed with the flexibility to adjust the number of modules according to the application in which it will be used. A smaller number of modules contributes to energy efficiency (Table 3).

The modular design of the robot allows for easy customization and adaptation to specific tasks (Table 4) and operational requirements. Modules such as arms, end effectors, sensors, and control units can be quickly replaced and reconfigured, enhancing the flexibility of its functionality.

### 2.3. Testing Station

**Figure 2:**
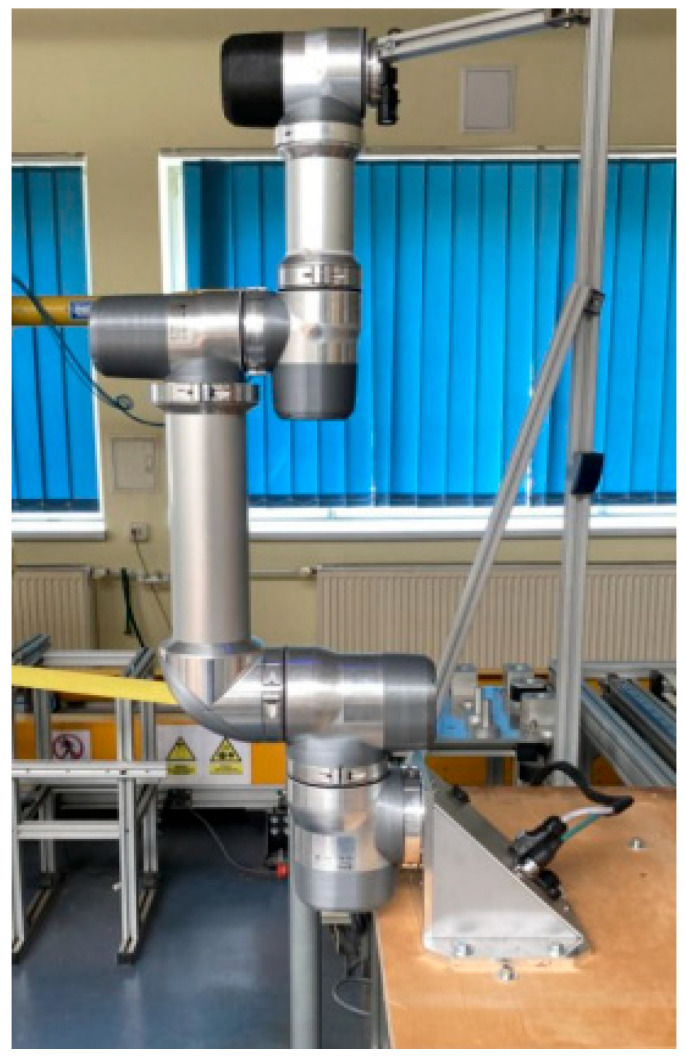
Testing station with mounted robot.

The testing station (Figure 2) was designed to minimize the impact of vibrations and other external factors on the operation of the robot and measuring devices. The station was built using aluminum profiles and a wooden plate. The structure was anchored to the floor.

**Figure 3:**
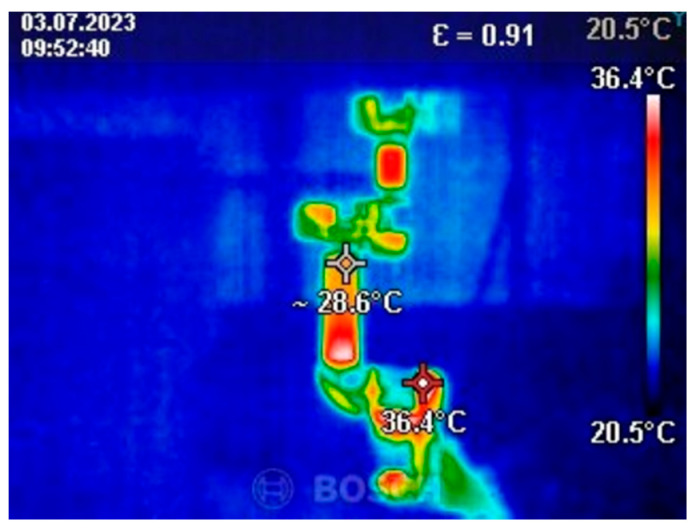
Thermal camera image.

Before starting the tests, programs with various applications were developed. The first was a program designed to heat the robot to its operating temperature. It involved simultaneous movement of all the robot’s axes to eliminate potential inaccuracies caused by temperature changes during operation. The program consisted of three blocks: a start block, an infinite loop, and a motion block comprising two positions. The robot was warmed up for 15 min immediately prior to testing. Below is an image of the temperature measurement taken after the warm-up process (Figure 3).

**Figure 4:**
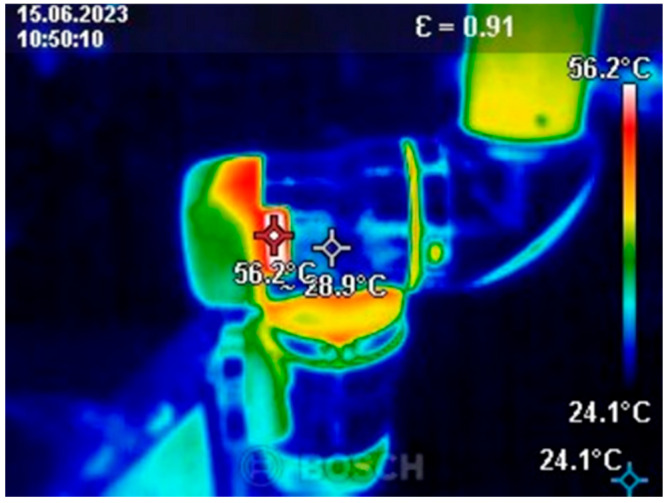
Maximum recorded temperature during testing.

The maximum measured temperature of the robot module was 56.2 °C (Figure 4). This temperature was recorded on the first robot module. The ambient temperature was 24 °C. The measurement was taken during robot testing.

### 2.4. Accuracy Testing of Selected Configurations

Positioning accuracy testing was conducted with three loads in the form of carbon steel blocks of varying masses: 5603.5 g, 9630.4 g, and 15,607.74 g. The weight of the blocks was supplemented by the mass of fastening screws: 75 g for 4 screws securing the 6 kg and 10 kg weights, and 135 g for 7 screws securing the 16 kg load. Additionally, an aluminum flange weighing 240 g was used. The total weight, including screws and flange, was 5918.5 g, 9945.4 g, and 15,922.74 g, respectively. For simplicity, the loads will be referred to as 6 kg, 10 kg, and 16 kg in the text. The purpose of using different loads was to examine their impact on the robot’s accuracy and repeatability.

**Figure 5:**
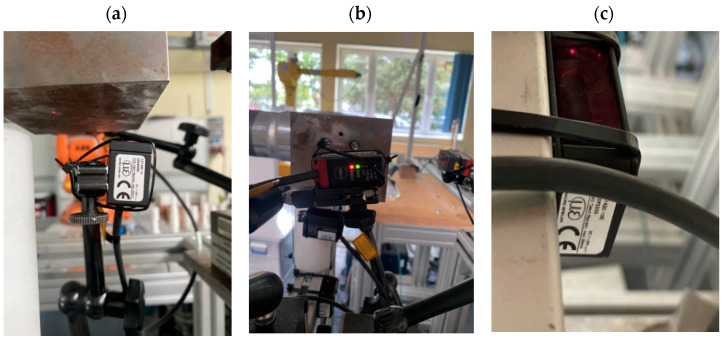
Optical sensors mounted on the testing station.

For the purpose of this study, four optical sensors from Micro-Epsilon company from USA, model optoNCDT ILD1420, with different measurement ranges were mounted on the testing station:

- IDL1420—10 (Figure 5a);
- IDL1420—50 (Figure 5b);
- IDL1420—100 (Figure 5c).

Selected technical parameters of the sensors are presented in Table 5.

**Figure 6:**
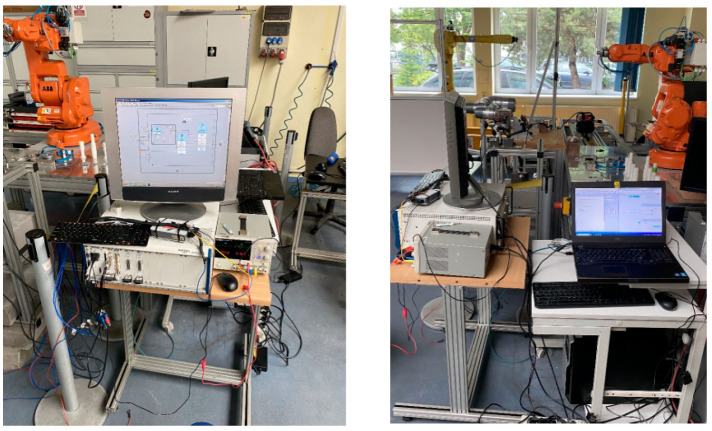
Industrial computer with peripherals.

The ILD1420-100 sensor, mounted underneath the structure (Figure 5b), served as a measurement trigger. When the robot’s end effector with the attached weight passed through the sensor’s beam within its measurement range, it triggered the measurement. Two ILD1420-50 sensors measured deviations and the position of the end effector along the tool’s X- and Y-axes. The ILD1420-10 sensor measured along the *Z*-axis. The sensors were connected to a data acquisition (DAQ) module, specifically the NI PXIe module, installed in a PXIe-1075 chassis. The entire setup was powered by a GW Instek GPD laboratory power supply. The station also included a monitor and a laptop connected to the robot (Figure 6).

### 2.5. Four-, Five-, and Six-Axis Configuration

**Figure 7:**
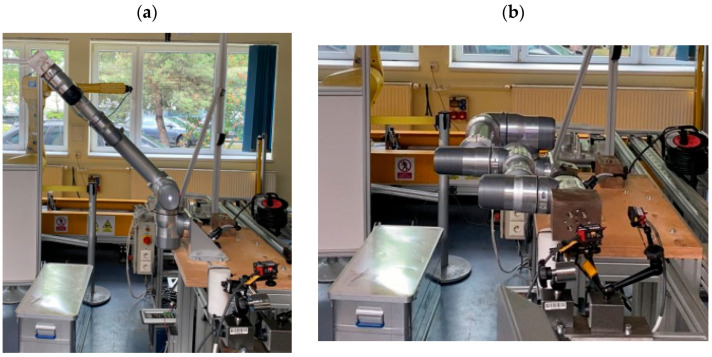
Starting position (**a**) and the robot’s first measurement position (**b**).

The initial measurements were conducted in a 6-axis configuration with a 6 kg load. The robot was positioned in its first starting position (Figure 7a). Then, it performed an axial movement, reaching the measurement position (Figure 7b) and triggering the measurement. The robot remained in the measurement position for 5.5 s.

**Figure 8:**
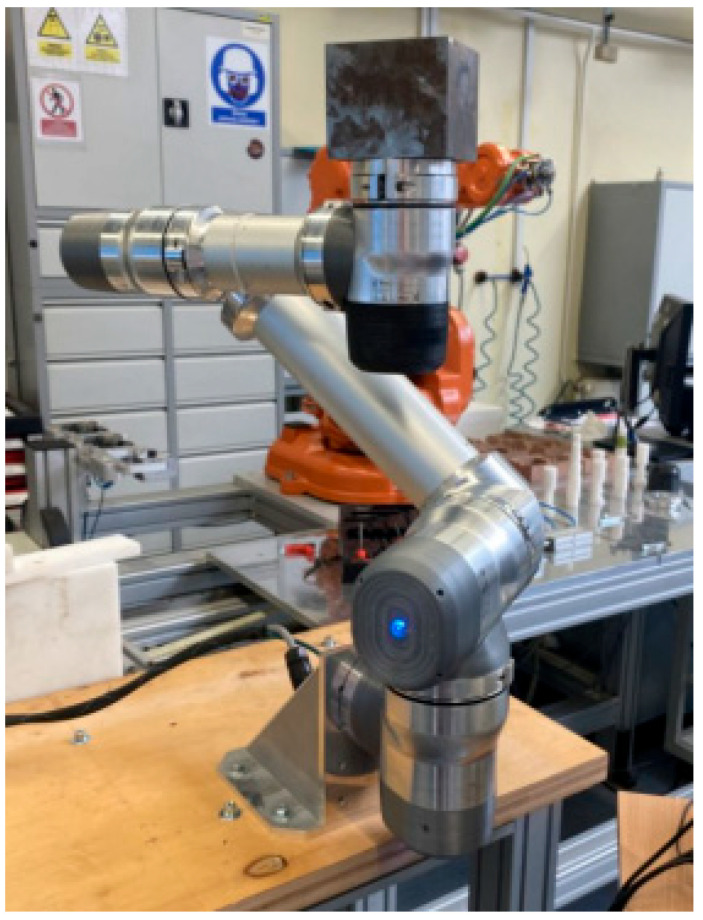
Service position.

The approach to the measurement position was repeated 30 times in accordance with the PN-EN ISO 9283 standard. The robot operated at a speed and acceleration set to 100%. After completing the measurements, the robot was placed in the service position (Figure 8), where the load was replaced.

**Figure 9:**
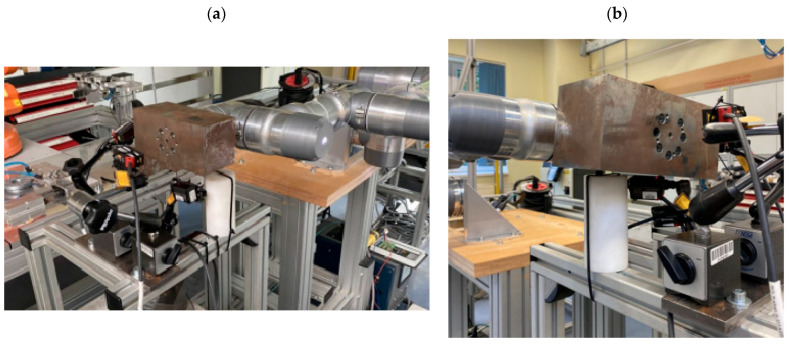
Measurement position of the robot with a 10 kg load (**a**) and a 16 kg load (**b**).

The test was repeated for a 10 kg load (Figure 9a) and a 16 kg load (Figure 9b).

**Figure 10:**
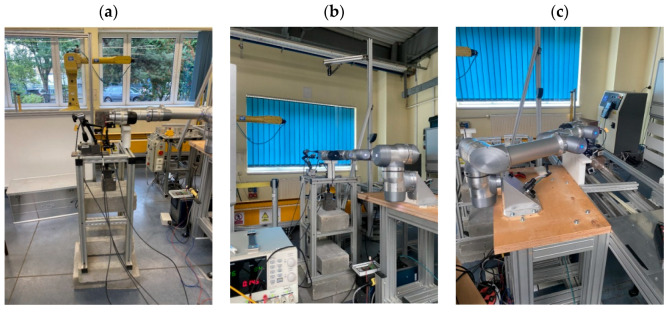
Measurement position of the robot: II (**a**), III (**b**), and IV (**c**).

In the 6-axis configuration, measurement with a 16 kg load proved impossible. The robot was unable to reach and maintain the target position. Reducing operating parameters did not improve the robot’s performance. After completing tests in Position I, the tests were repeated in the same manner for Positions II, III, and IV (Figure 10a–c).

Positions I, II, and III were offset by 90 degrees relative to each other on the robot’s first axis. Due to the robot’s limitations, Position IV could not be offset from Position III by the same value. Therefore, a 70-degree offset was set instead.

**Figure 11:**
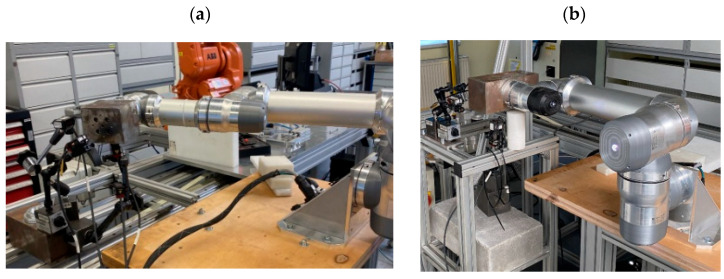
Robot in the measurement position with five modules (**a**) and four modules (**b**).

After completing measurements in the 6-module configuration, the robot was returned to the service position, reconfigured, and the tests described above were conducted with five modules (Figure 11a) and four modules (Figure 11b).

With five modules, the robot was able to repeatedly reach the target positions at maximum operating parameters with both the 6 kg and 10 kg loads. However, when a 16 kg load was mounted, the robot could not consistently reach the measurement positions. In the 4-module configuration, the robot was able to reliably reach the target position with every load.

## 3. Results

**Figure 12:**
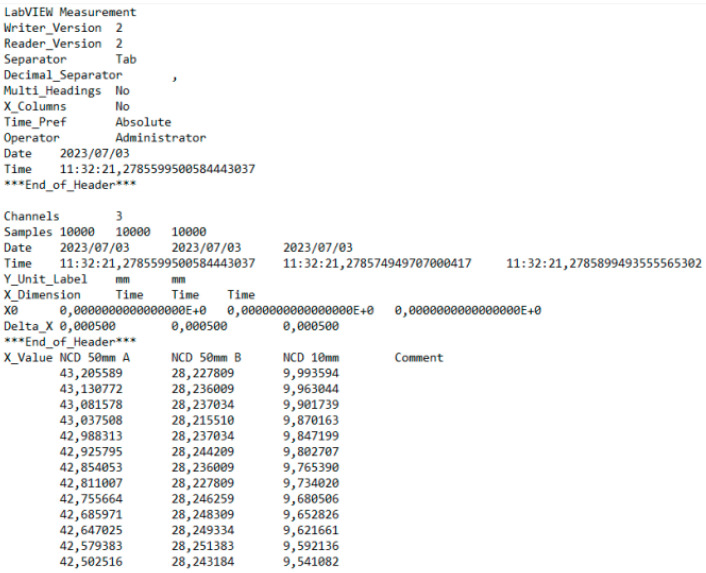
Excerpt from the LabVIEW program report.

During the accuracy testing of the robot, data were collected from three sensors across various configurations and loads. Each measurement lasted 5 s, during which 10,000 individual readings were gathered per sensor (Figure 12). Data were collected from 30 approaches for each position.

In the data analysis process, it was decided to select 30 readings from the center of the range of collected data for each measurement (10.000 values). These readings were within ±15 of the 5000th measurement (Table 6). After selecting the 30 readings, their average was calculated and used for further data analysis.

This average value was recorded in a table. The NCD 50 A sensor measured deviations close to the *X*-axis, NCD 50 B measured those close to the *Y*-axis, while NCD 10 measured deviations along the *Z*-axis. Based on the calculated data, graphs were created for each robot configuration.

The process was then repeated for each iteration of the test.

**Figure 13:**
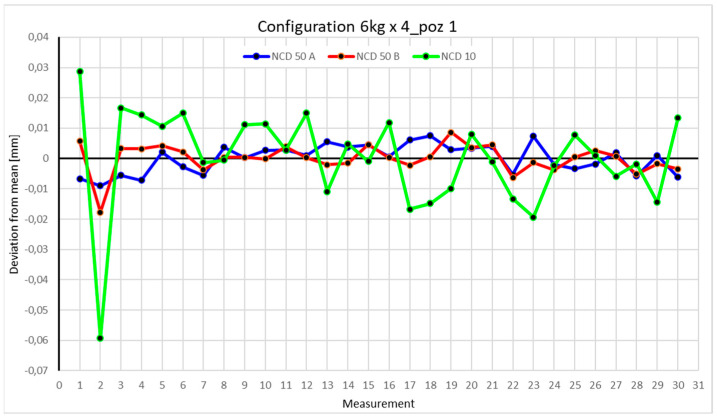
Chart for the 6 kg configuration with four modules in the first measurement position.

**Figure 14:**
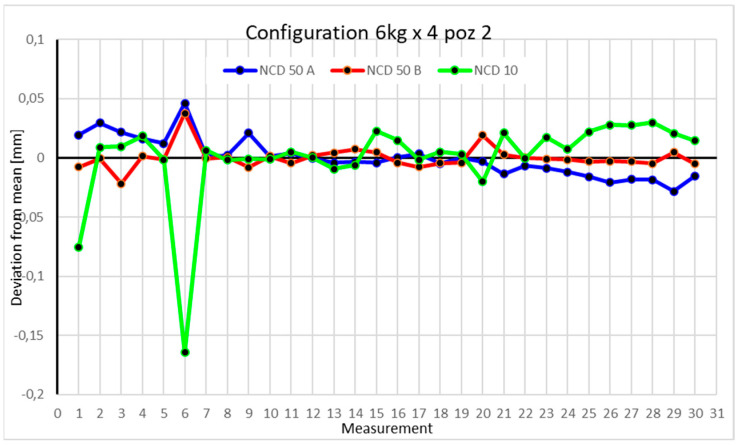
Chart for 6 kg configuration with four modules in the second measurement position.

**Figure 15:**
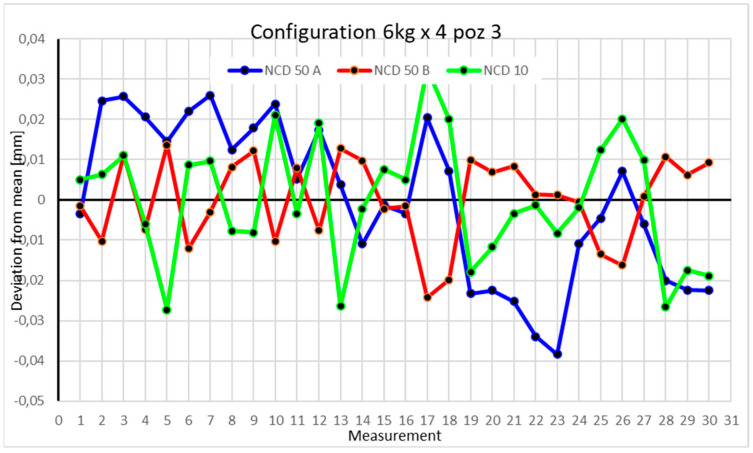
Chart for 6 kg configuration with four modules in the third measurement position.

**Figure 16:**
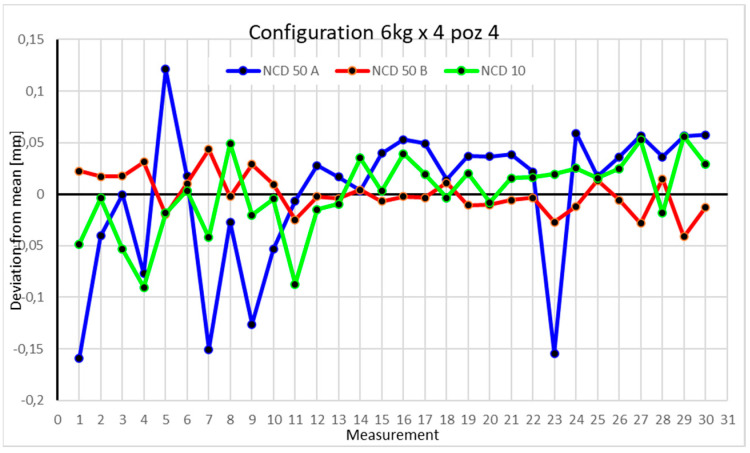
Chart for 6 kg configuration with four modules in Position IV.

Statistical data and data analysis for the four-module configuration with a 6 kg load (Figure 13, Figure 14, Figure 15 and Figure 16, Table 7, Table 8, Table 9 and Table 10).

The deviations for Position I did not exceed 0.1 mm. The range of measurements for the *X*-axis is 0.0165 mm, for the *Y*-axis 0.0264 mm, and for the *Z*-axis 0.0879 mm. The positive kurtosis indicates data concentration around the mean, as shown in the chart. The largest data scatter occurs with measurements from the NCD 10 sensor (*Z*-axis). The dataset is characterized by very low coefficients of variation: 0.013% for the *X*-axis, 0.017% for the *Y*-axis, and 0.226% for the *Z*-axis.

For Position II, deviations for the X- and Y-axes did not exceed 0.05 mm. For the *Z*-axis, two measurements fell within a range of 0.15 mm, while the rest were close to 0.05 mm. The range of measurements is 0.0742 mm for the *X*-axis, 0.0456 mm for the *Y*-axis, and 0.1943 mm for the *Z*-axis. Positive kurtosis is well visualized on the chart, showing that most data points concentrate around the mean. The largest scatter is observed for the NCD 10 sensor. The coefficients of variation are 0.084% for the *X*-axis, 0.043% for the *Y*-axis, and 1.091% for the *Z*-axis.

Position III is characterized by deviations smaller than 0.04 mm. The most significant changes occur for the NCD 50 A sensor. The coefficients of variation are 0.118% for the *X*-axis, 0.024% for the *Y*-axis, and 0.218% for the *Z*-axis. The measurement range does not exceed 0.08 mm. Negative kurtosis indicates a flattening of the normal distribution, with data values clustering more toward the extremes than the mean.

In Position IV, the largest scatter is observed in values collected by the NCD 50 A sensor, with a range of 0.280068 mm. The range for the *Y*-axis is 0.084354 mm and for the *Z*-axis is 0.146333 mm. Kurtosis for the *Y*-axis indicates data accumulation at the extremes. The coefficients of variation of 0.178%, 0.043%, and 0.786% suggest low data dispersion.

**Figure 17:**
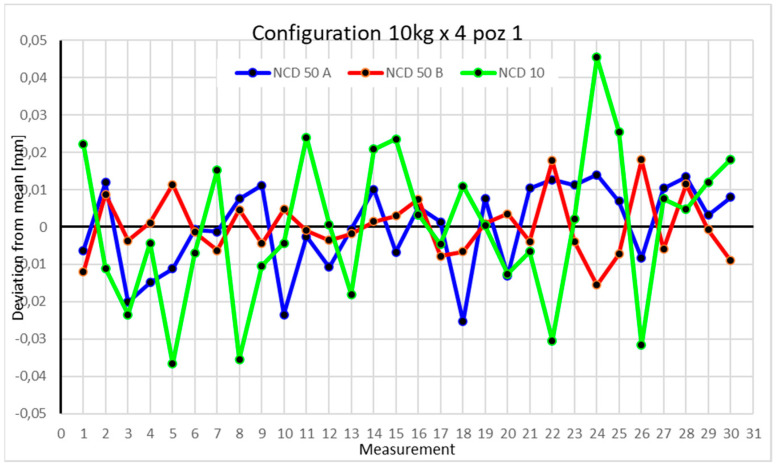
Chart for 10 kg configuration with four modules in the first measurement position.

**Figure 18:**
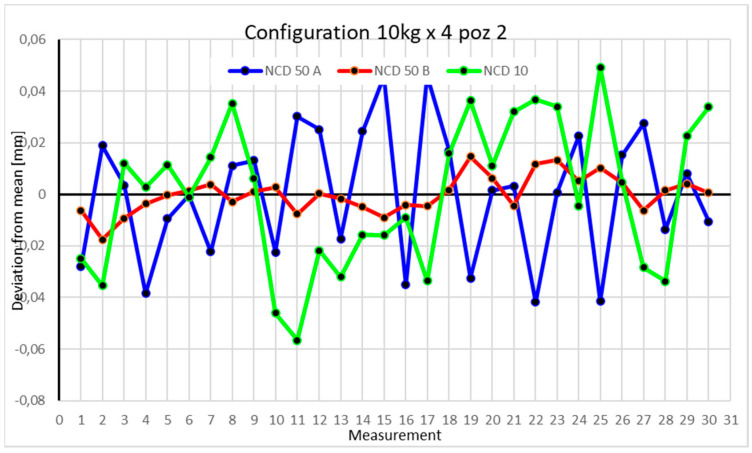
Chart for 10 kg configuration with four modules in the second measurement position.

**Figure 19:**
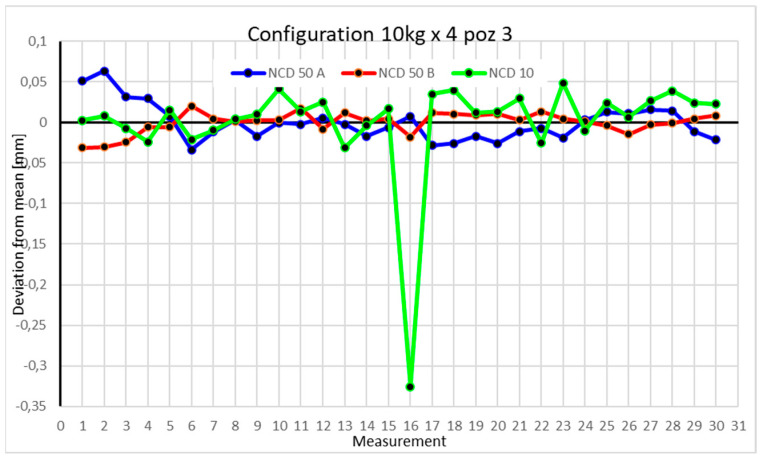
Chart for 10 kg configuration with four modules in the third measurement position.

**Figure 20:**
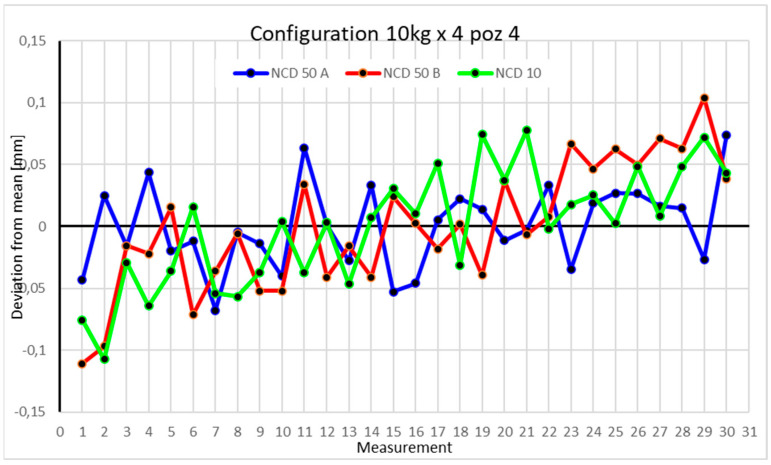
Chart for 10 kg configuration with four modules in the fourth measurement position.

Statistical data and analysis of the data collected for the four-module configuration with a 10 kg load (Figure 17, Figure 18, Figure 19 and Figure 20, Table 11, Table 12, Table 13 and Table 14).

For the first position with a 10 kg load and four modules, the deviation for the NCD 10 sensor exceeded −0.04 mm. Values for NCD 50 A surpassed −0.02 mm, while NCD 50 B ranged between −0.02 mm and 0.02 mm. The measurement range for the *X*-axis was 0.0393 mm, for the *Y*-axis 0.0335 mm, and for the *Z*-axis 0.0821 mm. The kurtosis values suggest data clustering around the extremes. The coefficients of variation are below 0.4%.

The second position displays a similar extremum, with values exceeding 0.04 mm on the X- and Z-axes. The range for the *X*-axis approaches 0.09 mm, while the *Z*-axis exceeds 0.1 mm. Both the coefficients of variation and kurtosis suggest strong clustering around extremes. The *Y*-axis shows a range of 0.03 mm, and kurtosis close to zero indicates data concentration near the mean.

In the third position, measurements for the *Y*-axis were closest to the mean. The X- and Z-axes exceeded 0.05 mm. For a single measurement, the deviation on the *Z*-axis was over 0.3 mm, potentially a measurement error. The rest of the values were closer to the mean. Low coefficients of variation and kurtosis suggest that data points are grouped near the mean for all measurements.

The fourth position shows larger deviations on the *Y*-axis compared to the X- and Z-axes, exceeding 0.01 mm. The ranges for the Z- and Y-axes are around 0.2 mm, while the *X*-axis is 0.15 mm. Negative kurtosis indicates that values deviate from the mean, with a tendency to spread toward the extremes.

**Figure 21:**
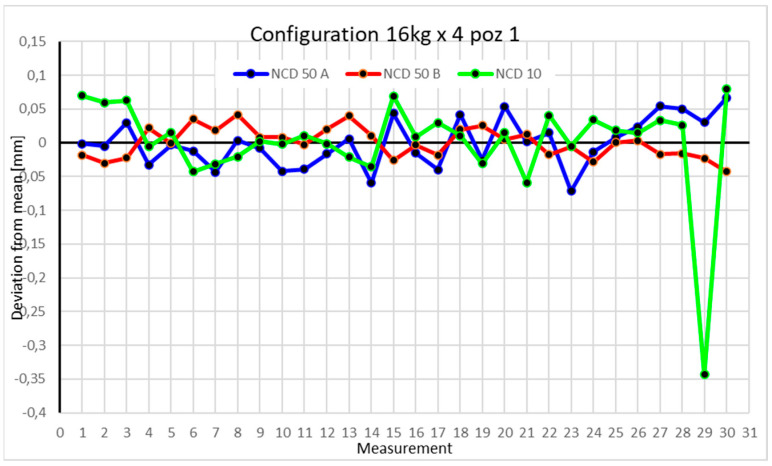
Chart for 16 kg configuration with four modules in the first measurement position.

**Figure 22:**
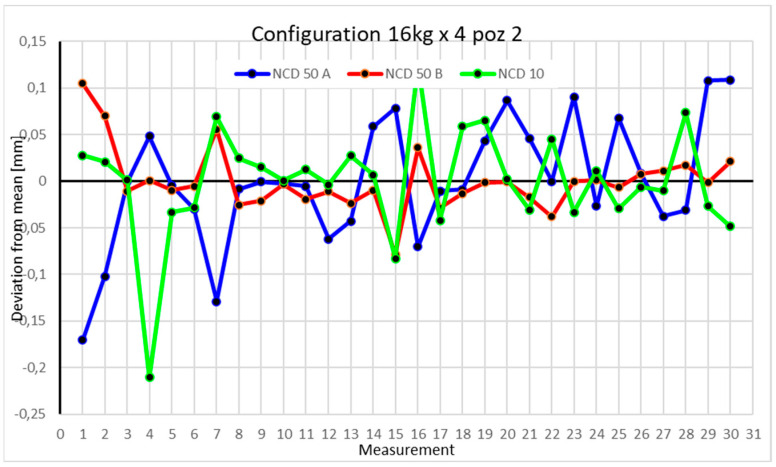
Chart for 16 kg configuration with four modules in the second measurement position.

**Figure 23:**
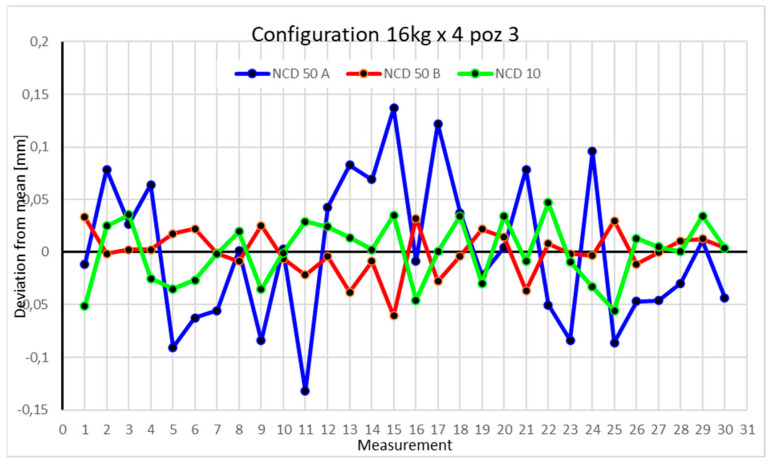
Chart for 16 kg configuration with four modules in the third measurement position.

**Figure 24:**
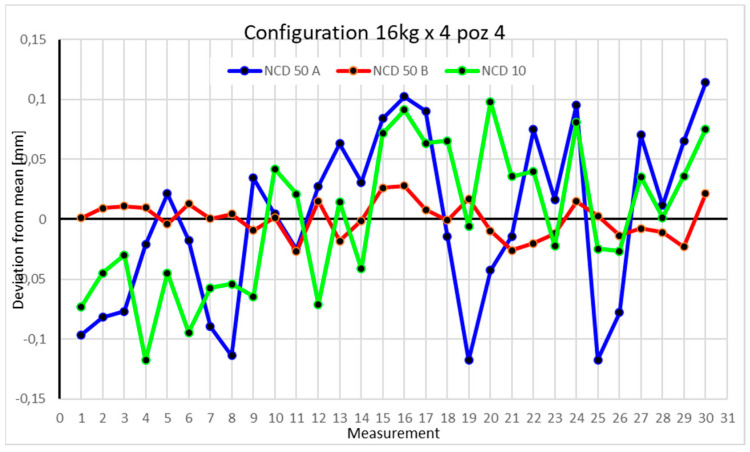
Chart for 16 kg configuration with four modules in the fourth measurement position.

Statistical data and analysis of the data collected for the four-module configuration with a 16 kg load (Figure 21, Figure 22, Figure 23 and Figure 24, Table 15, Table 16, Table 17 and Table 18).

For the first position with a 16 kg load, the NCD 10 sensor recorded one measurement significantly deviating from the mean, with a value of −0.3 mm from the mean. The rest of the measurements ranged from 0.1 mm to −0.1 mm from the mean. The kurtosis and coefficient of variation values indicate data concentration near the extremes.

In the second position, the kurtosis values are positive. Measurements from the NCD 50B sensor are the closest to the mean. The *Z*-axis values exhibit the highest coefficient of variation.

The third position is characterized by the greatest scatter on the X- and Z-axes, while the *Y*-axis values are the closest to the mean.

In the fourth position, there is a noticeable difference in the spread of values among the X-, Z-, and Y-axes. The lowest coefficient of variation is observed for the NCD 50B sensor.

**Figure 25:**
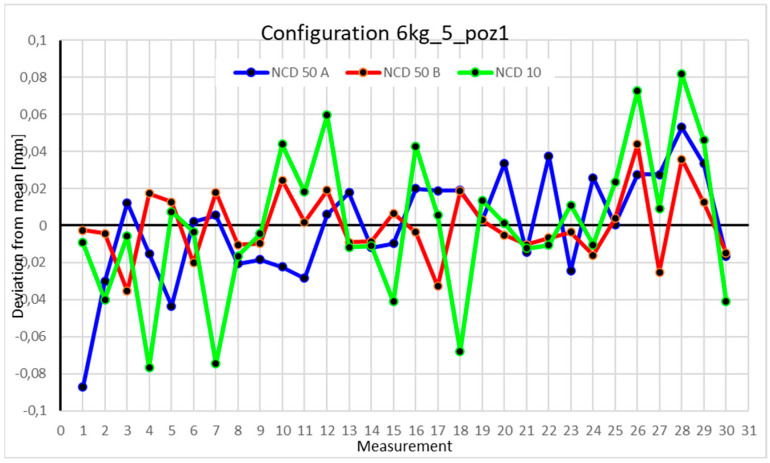
Chart for 6 kg configuration with five modules in the first measurement position.

**Figure 26:**
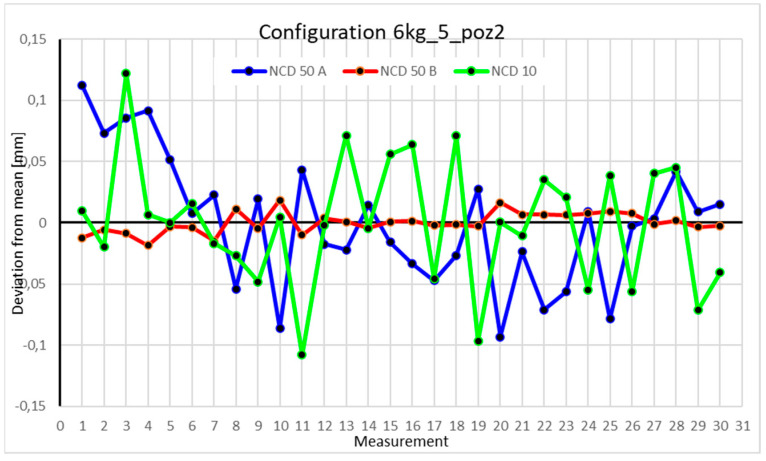
Chart for 6 kg configuration with five modules in the second measurement position.

**Figure 27:**
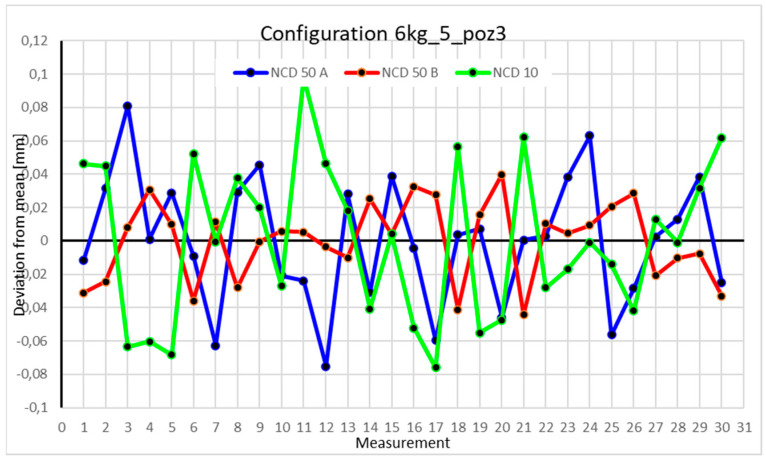
Chart for 6 kg configuration with five modules in the third measurement position.

**Figure 28:**
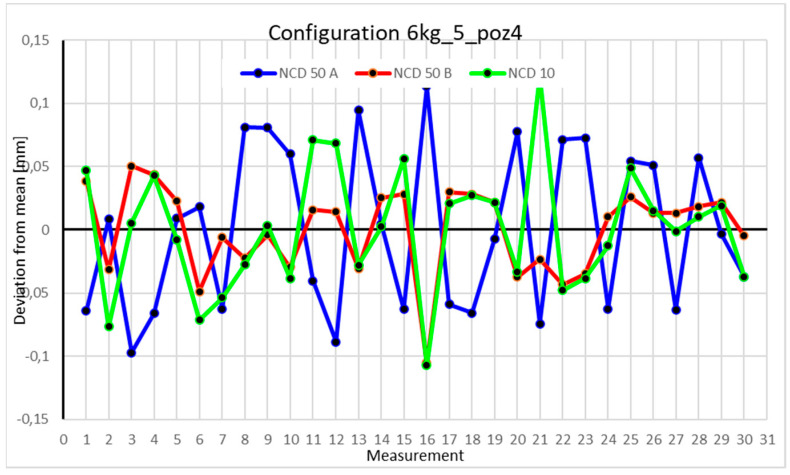
Chart for 6 kg configuration with five modules in the fourth measurement position.

Statistical data and analysis of the data collected for the five-module configuration with a 6 kg load (Figure 25, Figure 26, Figure 27 and Figure 28, Table 19, Table 20, Table 21 and Table 22).

**Figure 29:**
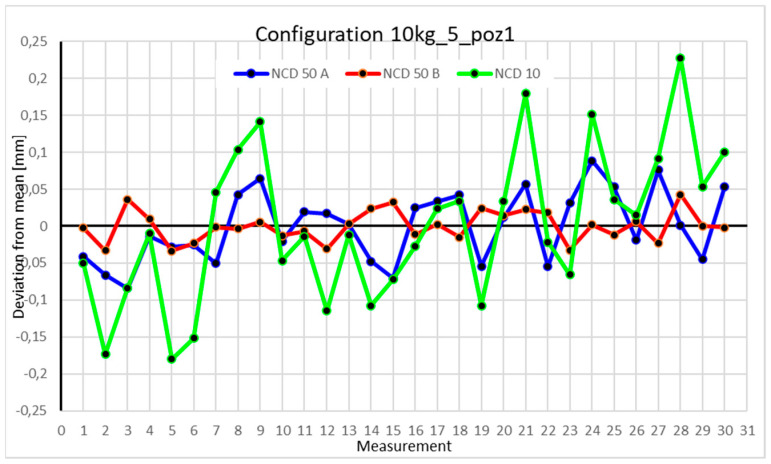
Chart for 10 kg configuration with five modules in the first measurement position.

**Figure 30:**
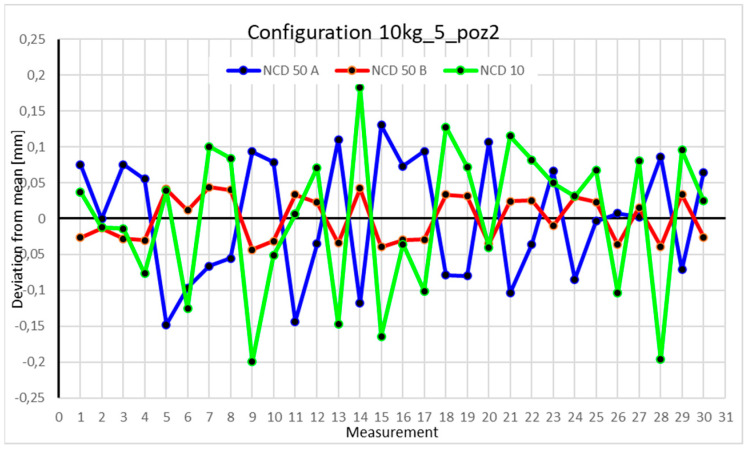
Chart for 10 kg configuration with five modules in the second measurement position.

**Figure 31:**
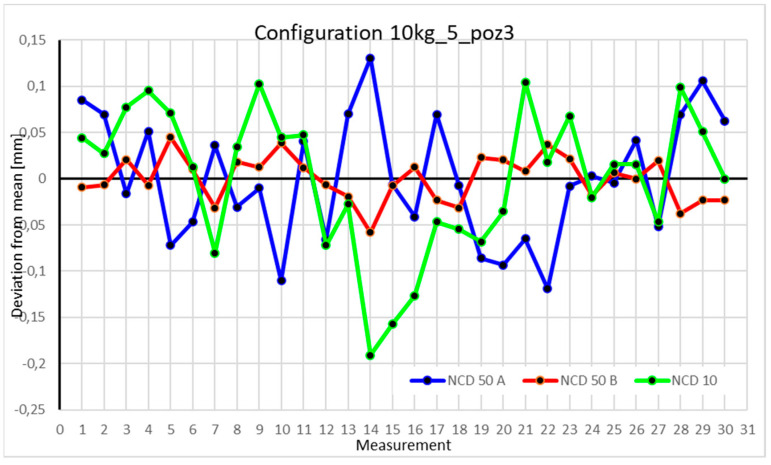
Chart for 10 kg configuration with five modules in the third measurement position.

**Figure 32:**
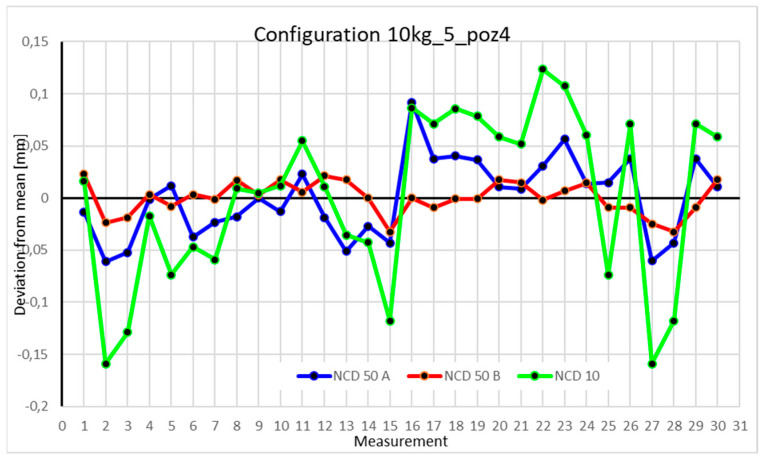
Chart for 10 kg configuration with five modules in the fourth measurement position.

Statistical data and analysis of the data collected for the five-module configuration with a 10 kg load (Figure 29, Figure 30, Figure 31 and Figure 32, Table 23, Table 24, Table 25 and Table 26).

**Figure 33:**
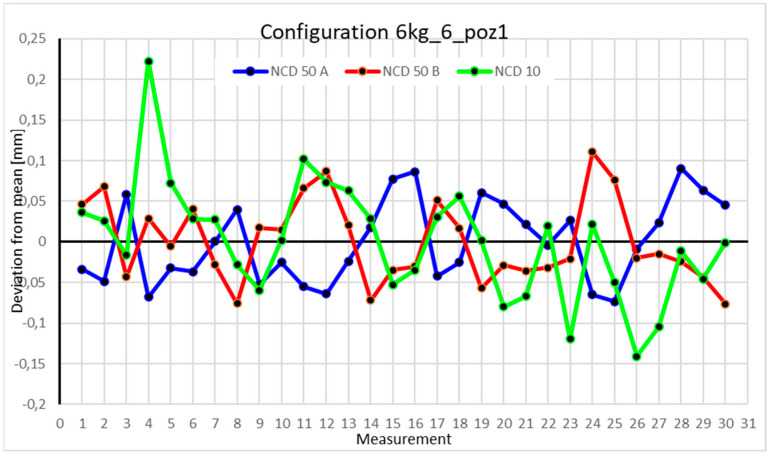
Chart for 6 kg configuration with six modules in the first measurement position.

**Figure 34:**
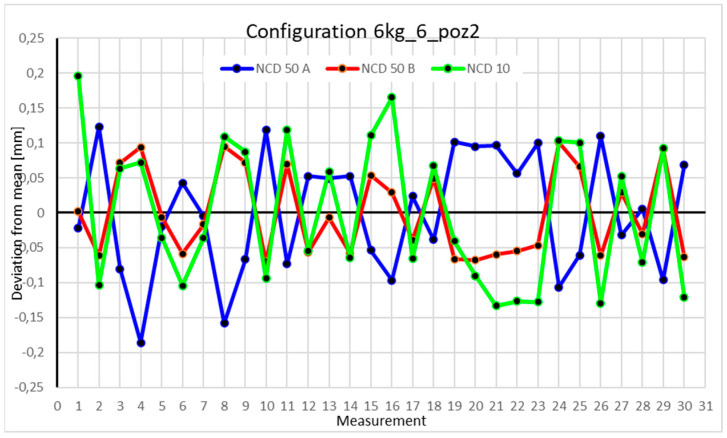
Chart for 6 kg configuration with six modules in the second measurement position.

**Figure 35:**
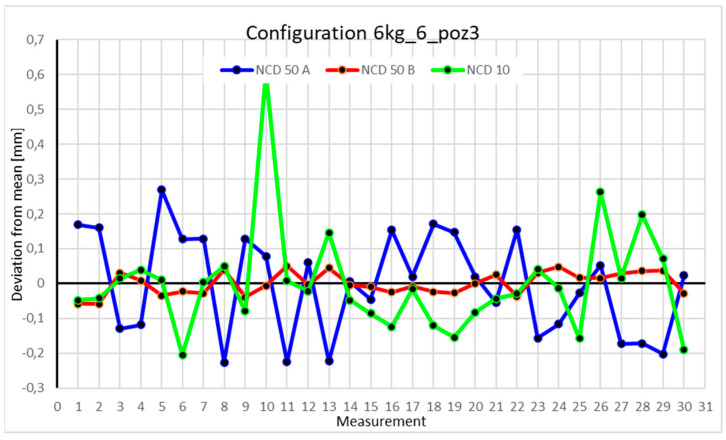
Chart for 6 kg configuration with six modules in the third measurement position.

**Figure 36:**
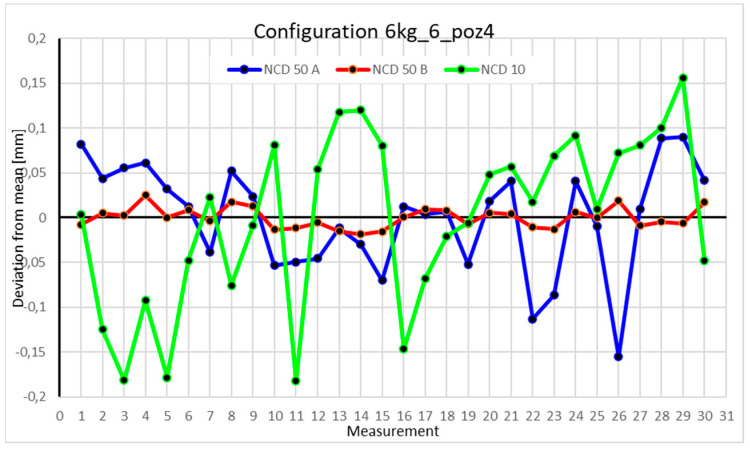
Chart for 6 kg configuration with six modules in the fourth measurement position.

Statistical data and analysis of the data collected for the six-module configuration with a 6 kg load (Figure 33, Figure 34, Figure 35 and Figure 36, Table 27, Table 28, Table 29 and Table 30).

## 4. Discussion

### 4.1. Analysis of the Impact of Load Variation on the Four-Module Configuration

Comparing the charts for Position I, a noticeable difference in deviations from the mean can be observed depending on the load. For the 6 kg load (Figure 13), most points, except for two outliers, are concentrated between −0.02 and 0.02. The points for the X- and Y-axes are clustered around the mean, while those for the *Z*-axis are more dispersed. For the 10 kg load (Figure 17), the *Z*-axis range increases significantly, frequently exceeding 0.02 and −0.02, with four points approaching −0.04. The points are also much more scattered for the *Z*-axis, as well as the X- and Y-axes, whose ranges have also increased. The 16 kg load (Figure 21) shows the most significant changes. The chart scale has changed, with some points oscillating around 0.07 for the Z- and X-axes. The largest deviations are observed on the *Z*-axis. For Position II, a similar trend is evident: as the load increases, the range expands, and the clustering around the mean decreases. With 6 kg (Figure 14), most values remain close to 0, not exceeding 0.025 and −0.025. With 10 kg (Figure 18), values fluctuate around 0.04 and −0.04, while for 16 kg (Figure 22), deviations reach around 0.1 and −0.05. The charts clearly show that load variations have a more significant impact on the fluctuations in the X- and Z-axes compared to the *Y*-axis. In Position III, the same pattern occurs. Deviations from the mean range from 0.03 to −0.03 for 6 kg (Figure 15), and from 0.05 to −0.05 for 10 kg (Figure 19). For 16 kg (Figure 23), the most significant changes are observed on the *X*-axis, where values approach 0.1 and −0.1, exceeding this range in three instances. Deviations for the Z- and Y-axes are similar to those for 10 kg but slightly larger. Analyzing the charts for Position IV, for 6 kg (Figure 16), large fluctuations in the *X*-axis are evident, greater than those observed for 16 kg (Figure 23) and 10 kg. For 10 kg (Figure 20), deviations for the Y- and Z-axes fall within a broader range than for 6 kg. For 16 kg, the range is similar, but the points are closer to the extremes.

### 4.2. Analysis of the Impact of Load Variation on the Five-Module Configuration

In the five-module configuration with a 6 kg load (Figure 20), the robot’s deviations from the mean ranged between −0.075 and 0.075 for the *Z*-axis, and ±0.05 for the X- and Y-axes. The *Y*-axis showed the smallest fluctuations. With a 10 kg load (Figure 29), the range for the Z- and X-axes was nearly twice as large as in the 6 kg configuration. *Z*-axis values approached −0.15 and 0.15, with occasional exceedances. The *X*-axis range for most measurements was around −0.05 to 0.05, while the *Y*-axis range also doubled to ±0.05. For the robot’s second position with a 6 kg load (Figure 26), a significant scatter was observed for the *X*-axis. Some values fluctuated between −0.1 and 0.1, while others stayed closer to the mean around ±0.05. The *Y*-axis remained concentrated near the mean, with a range not exceeding ±0.02. The *Z*-axis showed high variability, with a range between −0.1 and 0.1. With a 10 kg load (Figure 30), most *X*-axis measurements clustered near the extremes, occasionally approaching the mean. *Y*-axis values were evenly distributed within −0.05 to 0.05, a range twice as large as with the 6 kg load. For the third position with a 6 kg load (Figure 27), the *X*-axis exhibited significant scatter around the mean, with a range exceeding ±0.075. Most values were concentrated within ±0.05. The *Y*-axis had the smallest range, approximately ±0.05, while *Z*-axis values were centered around −0.075. With a 10 kg load (Figure 31), *X*-axis deviations reached above 0.1 and below −0.1. Most *Z*-axis measurements clustered around the extremes of ±0.1, occasionally exceeding −0.2. *Y*-axis values remained within ±0.05 of the mean. In the fourth approach position with a 6 kg load (Figure 28), all axes showed greater fluctuations compared to previous measurements. Most *X*-axis values ranged between 0.05 and 0.1 on both sides of the zero axis, and the same applied to the *Z*-axis. *Y*-axis values were within −0.05 to 0.05. For 10 kg (Figure 32), the *Z*-axis showed the greatest scatter, with values exceeding −0.15 and 0.1. X- and *Y*-axis values were similar to those recorded for the 6 kg load.

### 4.3. Analysis of the Impact of Configuration Changes on the Robot’s Performance with a 10 kg Load

Comparing the two charts for Position I in the four-module and five-module configurations reveals significant differences in the ranges of measured values. Measurements for each axis in the five-module configuration (Figure 29) differ by an order of magnitude compared to their counterparts in the four-module configuration (Figure 17). The same trend is observed in Position II. The values for the five-module configuration (Figure 30) oscillate around an absolute value of 0.1–0.15 from the zero axis, whereas for the four-module configuration (Figure 18), the values are much closer to 0.02. For Position III, the ranges for the five-module configuration (Figure 30) are twice as large as those for the four-module configuration (Figure 18). Position IV is an exception, as the deviations for both the four-module (Figure 19) and five-module (Figure 31) configurations fall within similar ranges.

### 4.4. Analysis of the Impact of Configuration Changes on the Robot’s Performance with a 6 kg Load

Analyzing the charts for 6 kg in different configurations, changes in deviations from the mean can be observed. For Position I and the four-module configuration (Figure 13), values for the X- and Y-axes rarely exceeded ±0.015. Values for the *Z*-axis ranged between −0.02 and 0.02. In the five-module configuration (Figure 26), the range of values for the *Y*-axis increases and exceeds ±0.025. The *X*-axis data reach values three times higher than in the four-module measurement, while *Z*-axis values double. In the six-module configuration (Figure 34), a further doubling of values compared to the previous configuration is observed. Many measurements for each axis approach levels of 0.1 and −0.1. For the second and third positions, the same trend was observed. Changing from the four-module configuration (Figure 14 and Figure 15) to the five-module configuration (Figure 27 and Figure 28) results in doubled values. Then, changing from five to six modules (Figure 35 and Figure 36) repeats this pattern. An exception is Position IV reached by the robot. In this case, the change in values is not as dramatic, and the values for four modules (Figure 16) are close to those recorded during operation with five (Figure 29) and six modules (Figure 36). Deviations for six modules reach the highest values.

The results of this study offer substantial practical insights for industries utilizing modular robots. The relationship between module configuration, load capacity, and positioning accuracy provides actionable guidelines for optimizing robotic performance in real-world settings. Firstly, manufacturing industries that require high precision in tasks such as assembly, welding, or inspection should prioritize configurations with fewer modules when handling lighter loads. This approach minimizes errors, reduces system complexity, and enhances repeatability. Secondly, industries dealing with variable load conditions, such as logistics or palletizing, can benefit from adaptive reconfiguration of robot modules. For instance, a reconfiguration strategy that balances the number of modules with load requirements can maintain operational accuracy while optimizing energy consumption.

Furthermore, the findings highlight the potential for integrating modular robots into Industry 4.0 frameworks. Their adaptability to dynamic production lines makes them ideal for smart manufacturing environments. The ability to reconfigure robots for diverse tasks without substantial hardware modifications aligns well with the growing trend of mass customization in production. The scalability of modular robots makes them suitable for businesses aiming to reduce operational costs. By optimizing module configurations based on task-specific needs, industries can lower energy consumption, prolong equipment life, and improve overall efficiency.

## 5. Conclusions

Comparison of charts and statistical data revealed changes in positioning accuracy depending on the weight mounted on the robot and the number of its modules. Comparing the data collected for four positions, with a 6 kg load, adding one module doubled the range of accuracy-related data. With six modules, the robot demonstrated worse repeatability than with four or five modules. The greatest fluctuations were observed in the *Z*-axis, which aligns with the direction of gravitational force. The *Y*-axis showed the smallest changes. It was also noted that working at the robot’s extreme range (position 4) impacted its repeatability. Deviations for this position were comparable to other positions in configurations with a higher number of modules. A similar situation was observed during tests on four- and five-module configurations with a 10 kg load. In this case, increasing the number of modules also increased deviations from the mean. Position IV again showed higher deviations compared to other positions. The impact on specific axes corresponded to the data collected during the 6 kg tests. When comparing data collected for the four-module configuration with different loads (6 kg, 10 kg, and 16 kg), a significant impact of increased end-module weight on the positioning accuracy of the industrial robot was demonstrated. Increasing the weight caused the most substantial changes in the X- and Z-axes. The modular robot is suitable for tasks that do not require high precision, such as palletizing and material handling. This product is constantly evolving and being updated, so tests should be repeated after significant updates and structural modifications. Future research should examine the impact of the number of axes and load on power consumption and changes in module temperature during prolonged operation.

## Figures and Tables

**Table 1:** Modular robot configuration.

Degrees of freedom (DOFs)	From 1 to 8
Payload capacity	Up to 20 kg
Range	200 mm–2000 mm
Repeatability	±0.1 mm
Max speed	180°/s or 2 m/s
Workspace	±270°
Base area	191 mm × 175 mm
Power consumption	250 W
Materials	Aluminum, polypropylene
Ambient temperature	0–50 °C
Standards	10218-1; EN ISO 13849-1, PL d, Cat. 3

**Table 2:** Control unit parameters.

Dimensions	L 400 mm, W 200 mm, H 300 mm
Operating voltage	100–240 V AC/50–60 Hz; 48 V DC
Protection class	IP 54
Interfaces	8 × digital I/O, IO-Link, ethernet, Modbus TCP
Communication	sockets—RJ45
Programming	12′′ control panel, MATLAB, ROS, HTTP API

**Table 3:** Drive modules.

	M (D86)	L (D116)	XL (D148)
Diameter	86 mm	116 mm	148 mm
Nominal torque	29.7 Nm	55 Nm	120 Nm
Maximum torque	70 Nm	178 Nm	374 Nm
Nominal power	131 W	141 W	165 W
Maximum speed	230°/s	180°/s	180°/s
Protection class	IP 54	IP 54	IP 54

**Table 4:** Connecting modules.

Diameter	86 mm	116 mm	148 mm
Length type—I	200, 300, 400	300, 350, 600, 750	400, 600, 800
Length type—L	240, 340, 440	350, 500, 650, 800	470, 670, 870
Protection class	IP 54	IP 54	IP 54

**Table 5:** Parameters of ILD1420 sensors.

Model:	ILD1420-10	ILD1420-50	ILD1420-100
Range:	10 mm	50 mm	100 mm
Frequency:	4 kHz/2 kHz/1 kHz/0.5 kHz/0.25 kHz		
Linearity:	<±8 µm	<±0 µm	<±80 µm
Repeatability:	0.5 µm	2 µm	4 µm
Temperature range:	0–50 °C		
Range start:	20 mm	35 mm	50 mm
Mid-range:	25 mm	60 mm	100 mm
Range end:	30 mm	85 mm	150 mm

**Table 6:** Example table for determining values for further analysis.

6 kg × 4_1	NCD 50 mm A	NCD 50 mm B	NCD 10 mm
4985	37.71527	28.5353	7.199787
4986	37.70297	28.52607	7.199172
4987	37.72449	28.53222	7.199992
4988	37.71732	28.52095	7.197122
4989	37.70502	28.53837	7.200402
4990	37.71834	28.53017	7.199992
4991	37.70809	28.53325	7.200402
4992	37.72757	28.52095	7.197122
4993	37.70399	28.53427	7.200402
4994	37.71834	28.52402	7.199787
4995	37.71014	28.53427	7.199582
4996	37.71629	28.523	7.197327
4997	37.70502	28.52915	7.200812
4998	37.72142	28.5271	7.199787
4999	37.70912	28.52812	7.199582
5000	37.70297	28.52197	7.197737
5001	37.71424	28.5312	7.201223
5002	37.69887	28.5271	7.199992
5003	37.71117	28.53632	7.199787
5004	37.71322	28.5189	7.198147
5005	37.71834	28.53325	7.200607
5006	37.71629	28.52812	7.199787
5007	37.70399	28.53735	7.199992
5008	37.71117	28.51787	7.198147
5009	37.70604	28.53222	7.200197
5010	37.71834	28.5271	7.199992
5011	37.70502	28.53632	7.199582
5012	37.72142	28.5148	7.199172
5013	37.70707	28.53017	7.199992
5014	37.71527	28.53017	7.200812
Mean:	37.7122	28.5286	7.1995

**Table 7:** Statistical data based on measurements for a 6 kg load in the four-axis configuration in position 1.

	NCD 50 A	NCD 50 B	NCD 10
Mean [mm]	37.718883	28.5229	7.1709
Standard error [mm]	0.0008677	0.00088	0.00296
Median [mm]	37.7199	28.5232	7.17115
First quartile [mm]	37.714075	28.5209	7.162
Third quartile [mm]	37.722525	28.5261	7.18223
Variance [mm^2^]	2.259 × 10^5^	2.3 × 10^5^	0.00026
Standard deviation [mm]	0.0047524	0.0048	0.01623
Kurtosis	−1.131844	5.55451	5.06387
Skewness	−0.272817	−1.62746	−1.5958
Range [mm]	0.0165	0.0264	0.0879
Minimum [mm]	37.7099	28.5051	7.1116
Maximum [mm]	37.7264	28.5315	7.1995
Count	30	30	30
Coefficient of variation [%]	0.013%	0.017%	0.226%

**Table 8:** Statistical data based on measurements for a 6 kg load in the four-axis configuration in Position II.

	NCD 50 A	NCD 50 B	NCD 10
Mean [mm]	19.386	22.324	3.35854
Standard error [mm]	0.00296	0.00177	0.00669
Median [mm]	19.3842	22.323	3.3643
First quartile [mm]	19.3749	22.3196	3.3572
Third quartile [mm]	19.39	22.326	3.37675
Variance [mm^2^]	0.00026	9.4 × 10^5^	0.00134
Standard deviation [mm]	0.01623	0.00971	0.03663
Kurtosis	1.01207	8.05292	14.6828
Skewness	0.8566	1.95445	−3.56547
Range [mm]	0.0742	0.0595	0.1943
Minimum [mm]	19.3578	22.3021	3.1942
Maximum [mm]	19.432	22.3616	3.3885
Count	30	30	30
Coefficient of variation [%]	0.084%	0.043%	1.091%

**Table 9:** Statistical data based on measurements for a 6 kg load in the four-axis configuration in Position III.

	NCD 50 A	NCD 50 B	NCD 10
Mean [mm]	16.4438	43.3914	7.07147
Standard error [mm]	0.00356	0.00192	0.00281
Median [mm]	16.4451	43.3925	7.06983
First quartile [mm]	16.4261	43.3839	7.06321
Third quartile [mm]	16.4616	43.4005	7.08124
Variance [mm^2^]	0.00038	0.00011	0.00024
Standard deviation [mm]	0.01948	0.01054	0.01542
Kurtosis	−1.10221	−0.56311	−0.51898
Skewness	−0.30704	−0.61966	−0.04863
Range [mm]	0.0644	0.03786	0.05994
Minimum [mm]	16.4055	43.3672	7.04419
Maximum [mm]	16.4699	43.4051	7.10412
Count	30	30	30
Coefficient of variation [%]	0.118%	0.024%	0.218%

**Table 10:** Statistical data based on measurements for a 6 kg load in the four-axis configuration in Position IV.

	NCD 50 A	NCD 50 B	NCD 10
Mean [mm]	39.4326	44.4251	4.68563
Standard error [mm]	0.0128	0.00346	0.00672
Median [mm]	39.4521	44.4222	4.68882
First quartile [mm]	39.4107	44.4146	4.66817
Third quartile [mm]	39.472	44.4378	4.70928
Variance [mm^2^]	0.00492	0.00036	0.00136
Standard deviation [mm]	0.07011	0.01897	0.03682
Kurtosis	0.70679	0.04735	0.61147
Skewness	−1.14153	0.1311	−0.84248
Range [mm]	0.28007	0.08435	0.14633
Minimum [mm]	39.2735	44.384	4.59527
Maximum [mm]	39.5536	44.4684	4.7416
Count	30	30	30
Coefficient of variation [%]	0.178%	0.043%	0.786%

**Table 11:** Statistical data based on measurements for a 10 kg load in the four-axis configuration in Position I.

	NCD 50 A	NCD 50 B	NCD 10
Mean [mm]	3.05792	26.9764	7.7523
Standard error [mm]	0.00212	0.00147	0.00366
Median [mm]	3.06018	26.9752	7.75281
First quartile [mm]	3.04998	26.9708	7.7414
Third quartile [mm]	3.06818	26.9807	7.76677
Variance [mm^2^]	0.00013	6.5 × 10^−5^	0.0004
Standard deviation [mm]	0.0116	0.00805	0.02004
Kurtosis	−0.55366	0.16022	−0.26067
Skewness	−0.70295	0.55007	−0.03323
Range [mm]	0.03929	0.03348	0.08216
Minimum [mm]	3.0326	26.9609	7.71563
Maximum [mm]	3.07188	26.9943	7.79779
Count	30	30	30
Coefficient of variation [%]	0.379%	0.030%	0.259%

**Table 12:** Statistical data based on measurements for a 10 kg load in the four-axis configuration in Position II.

	NCD 50 A	NCD 50 B	NCD 10
Mean [mm]	9.97326	9.67728	6.47147
Standard error [mm]	0.00462	0.0013	0.00519
Median [mm]	9.97558	9.67771	6.47514
First quartile [mm]	9.95229	9.67275	6.44739
Third quartile [mm]	9.99169	9.68128	6.49241
Variance [mm^2^]	0.00064	5.1 × 10^−5^	0.00081
Standard deviation [mm]	0.02529	0.00714	0.02843
Kurtosis	−0.89171	0.34663	−0.97865
Skewness	−0.0721	0.04769	−0.14989
Range [mm]	0.08783	0.03215	0.10575
Minimum [mm]	9.93149	9.65979	6.41497
Maximum [mm]	10.0193	9.69194	6.52072
Count	30	30	30
Coefficient of variation [%]	0.254%	0.074%	0.439%

**Table 13:** Statistical data based on measurements for a 10 kg load in the four-axis configuration in Position III.

	NCD 50 A	NCD 50 B	NCD 10
Mean [mm]	23.8427	39.7367	7.30772
Standard error [mm]	0.00413	0.00236	0.01186
Median [mm]	23.8405	39.7394	7.3202
First quartile [mm]	23.8257	39.7314	7.30124
Third quartile [mm]	23.8529	39.7454	7.33265
Variance [mm^2^]	0.00051	0.00017	0.00422
Standard deviation [mm]	0.02262	0.01291	0.06496
Kurtosis	1.26157	0.67405	23.4414
Skewness	1.0467	−0.99441	−4.59365
Range [mm]	0.09695	0.05138	0.3742
Minimum [mm]	23.8091	39.7057	6.98222
Maximum [mm]	23.9061	39.7571	7.35643
Count	30	30	30
Coefficient of variation [%]	0.095%	0.032%	0.889%

**Table 14:** Statistical data derived from measurements for a 10 kg load in the four-axis configuration in Position IV.

	NCD 50 A	NCD 50 B	NCD 10
Mean [mm]	29.5057	48.3368	4.80955
Standard error [mm]	0.00625	0.00944	0.00866
Median [mm]	29.5046	48.3348	4.81498
First quartile [mm]	29.4806	48.2982	4.77281
Third quartile [mm]	29.53	48.375	4.84524
Variance [mm^2^]	0.00117	0.00268	0.00225
Standard deviation [mm]	0.03425	0.05172	0.04741
Kurtosis	−0.36531	−0.36457	−0.5586
Skewness	0.09534	−0.14058	−0.2701
Range [mm]	0.14178	0.21517	0.18473
Minimum [mm]	29.438	48.2258	4.70224
Maximum [mm]	29.5797	48.4409	4.88697
Count	30	30	30
Coefficient of variation [%]	0.116%	0.107%	0.986%

**Table 15:** Statistical data based on measurements for a 16 kg load in the four-axis configuration in Position I.

	NCD 50 A	NCD 50 B	NCD 10
Mean [mm]	31.9016	31.6216	8.50332
Standard error [mm]	0.00655	0.00402	0.01346
Median [mm]	31.8993	31.6213	8.51408
First quartile [mm]	31.8785	31.6038	8.48623
Third quartile [mm]	31.9297	31.6386	8.53588
Variance [mm^2^]	0.00129	0.00049	0.00544
Standard deviation [mm]	0.03585	0.02204	0.07373
Kurtosis	−0.65458	−0.71946	16.8252
Skewness	0.04362	0.1352	−3.60037
Range [mm]	0.13856	0.08463	0.42291
Minimum [mm]	31.83	31.5789	8.15993
Maximum [mm]	31.9686	31.6635	8.58284
Count	30	30	30
Coefficient of variation [%]	0.112%	0.070%	0.867%

**Table 16:** Statistical data based on measurements for a 16 kg load in the four-axis configuration in Position II.

	NCD 50 A	NCD 50 B	NCD 10
Mean [mm]	29.625	13.2753	6.97267
Standard error [mm]	0.01225	0.00613	0.01071
Median [mm]	29.621	13.2709	6.97421
First quartile [mm]	29.5942	13.2591	6.9437
Third quartile [mm]	29.6727	13.2813	6.99913
Variance [mm^2^]	0.0045	0.00113	0.00344
Standard deviation [mm]	0.0671	0.03359	0.05864
Kurtosis	0.33195	3.29499	5.14502
Skewness	−0.45496	1.08422	−1.27299
Range [mm]	0.27891	0.18357	0.33591
Minimum [mm]	29.4547	13.1967	6.76201
Maximum [mm]	29.7336	13.3802	7.09792
Count	30	30	30
Coefficient of variation [%]	0.227%	0.253%	0.841%

**Table 17:** Statistical data based on measurements for a 16 kg load in the four-axis configuration in Position III.

	NCD 50 A	NCD 50 B	NCD 10
Mean [mm]	32.9311	30.2359	7.74041
Standard error [mm]	0.01265	0.00395	0.00534
Median [mm]	32.9274	30.2352	7.74191
First quartile [mm]	32.8816	30.2278	7.71402
Third quartile [mm]	32.9898	30.2496	7.76506
Variance [mm^2^]	0.0048	0.00047	0.00086
Standard deviation [mm]	0.06931	0.02161	0.02927
Kurtosis	−0.77444	0.97815	−0.99284
Skewness	0.19653	−0.78529	−0.30699
Range [mm]	0.26903	0.09365	0.10226
Minimum [mm]	32.7989	30.1754	7.68494
Maximum [mm]	33.0679	30.2691	7.78721
Count	30	30	30
Coefficient of variation [%]	0.210%	0.071%	0.378%

**Table 18:** Statistical data based on measurements for a 16 kg load in the four-axis configuration in Position IV.

	NCD 50 A	NCD 50 B	NCD 10
Mean [mm]	21.3359	34.9952	5.5432
Standard error [mm]	0.01315	0.00275	0.0109
Median [mm]	21.3439	34.9959	5.54094
First quartile [mm]	21.2676	34.9847	5.49837
Third quartile [mm]	21.4006	35.0058	5.58471
Variance [mm^2^]	0.00519	0.00023	0.00357
Standard deviation [mm]	0.07204	0.01508	0.05973
Kurtosis	−1.14673	−0.74126	−1.05913
Skewness	−0.17353	−0.04443	−0.05274
Range [mm]	0.23173	0.0548	0.2157
Minimum [mm]	21.2182	34.9684	5.42549
Maximum [mm]	21.45	35.0232	5.64119
Count	30	30	30
Coefficient of variation [%]	0.338%	0.043%	1.077%

**Table 19:** Statistical data based on measurements for a 6 kg load in the five-axis configuration in Position I.

	NCD 50 A	NCD 50 B	NCD 10
Mean [mm]	38.7099	28.965	7.67354
Standard error [mm]	0.00529	0.00338	0.00718
Median [mm]	38.7122	28.9613	7.66963
First quartile [mm]	38.692	28.9548	7.66139
Third quartile [mm]	38.7296	28.9777	7.69051
Variance [mm^2^]	0.00084	0.00034	0.00155
Standard deviation [mm]	0.02895	0.01852	0.03933
Kurtosis	1.4106	0.15006	0.07969
Skewness	−0.74179	0.34068	0.02251
Range [mm]	0.1402	0.07933	0.1584
Minimum [mm]	38.6226	28.9296	7.59699
Maximum [mm]	38.7628	29.0089	7.7554
Count	30	30	30
Coefficient of variation [%]	0.075%	0.064%	0.512%

**Table 20:** Statistical data based on measurements for a 6 kg load in the five-axis configuration in Position II.

	NCD 50 A	NCD 50 B	NCD 10
Mean [mm]	15.1622	21.4378	0.70221
Standard error [mm]	0.00967	0.00156	0.00954
Median [mm]	15.1679	21.4364	0.70246
First quartile [mm]	15.1305	21.4338	0.66509
Third quartile [mm]	15.1885	21.4443	0.74
Variance [mm^2^]	0.00281	7.3 × 10^−5^	0.00273
Standard deviation [mm]	0.05299	0.00852	0.05227
Kurtosis	−0.39576	0.11389	0.01963
Skewness	0.17837	0.08537	0.01935
Range [mm]	0.20535	0.03676	0.22999
Minimum [mm]	15.069	21.4195	0.59426
Maximum [mm]	15.2744	21.4562	0.82425
Count	30	30	30
Coefficient of variation [%]	0.349%	0.040%	7.444%

**Table 21:** Statistical data based on measurements for a 6 kg load in the five-axis configuration in Position III.

	NCD 50 A	NCD 50 B	NCD 10
Mean [mm]	13.1049	45.9177	2.29231
Standard error [mm]	0.00701	0.00431	0.00859
Median [mm]	13.1066	45.9227	2.29168
First quartile [mm]	13.0802	45.8997	2.25082
Third quartile [mm]	13.1341	45.9323	2.33538
Variance [mm^2^]	0.00148	0.00056	0.00222
Standard deviation [mm]	0.03841	0.02358	0.04708
Kurtosis	−0.42289	−0.85947	−1.01892
Skewness	−0.06478	−0.30853	0.10483
Range [mm]	0.15602	0.08391	0.1739
Minimum [mm]	13.0298	45.8736	2.21633
Maximum [mm]	13.1858	45.9575	2.39023
Count	30	30	30
Coefficient of variation [%]	0.293%	0.051%	2.054%

**Table 22:** Statistical data based on measurements for a 6 kg load in the five-axis configuration in Position IV.

	NCD 50 A	NCD 50 B	NCD 10
Mean [mm]	19.2403	40.1262	9.08838
Standard error [mm]	0.01185	0.00628	0.00906
Median [mm]	19.2409	40.1395	9.09129
First quartile [mm]	19.1775	40.0982	9.05231
Third quartile [mm]	19.2994	40.1508	9.11428
Variance [mm^2^]	0.00421	0.00118	0.00246
Standard deviation [mm]	0.06489	0.03439	0.04961
Kurtosis	−1.48252	1.34768	0.19464
Skewness	0.15099	−1.03601	0.12989
Range [mm]	0.21055	0.15569	0.2285
Minimum [mm]	19.1432	40.021	8.98108
Maximum [mm]	19.3538	40.1767	9.20958
Count	30	30	30
Coefficient of variation [%]	0.337%	0.086%	0.546%

**Table 23:** Statistical data based on measurements for a 10 kg load in the five-axis configuration in Position I.

	NCD 50 A	NCD 50 B	NCD 10
Mean [mm]	39.0596	31.6914	5.74858
Standard error [mm]	0.00878	0.00379	0.01889
Median [mm]	39.0616	31.6909	5.73755
First quartile [mm]	39.0158	31.6788	5.67816
Third quartile [mm]	39.0996	31.7044	5.80045
Variance [mm^2^]	0.00231	0.00043	0.0107
Standard deviation [mm]	0.04807	0.02078	0.10345
Kurtosis	−1.12169	−0.53584	−0.37427
Skewness	0.04533	0.15213	0.23922
Range [mm]	0.17252	0.07629	0.4078
Minimum [mm]	38.9756	31.6579	5.56867
Maximum [mm]	39.1481	31.7342	5.97647
Count	30	30	30
Coefficient of variation [%]	0.123%	0.066%	1.800%

**Table 24:** Statistical data based on measurements for a 10 kg load in the five-axis configuration in Position II.

	NCD 50 A	NCD 50 B	NCD 10
Mean [mm]	21.831	4.16627	7.86292
Standard error [mm]	0.01559	0.00583	0.01855
Median [mm]	21.8319	4.16711	7.8908
First quartile [mm]	21.7543	4.13619	7.7929
Third quartile [mm]	21.9065	4.19728	7.94155
Variance [mm^2^]	0.00729	0.00102	0.01032
Standard deviation [mm]	0.08539	0.03193	0.10161
Kurtosis	−1.38781	−1.83675	−0.66094
Skewness	−0.16755	0.02365	−0.46891
Range [mm]	0.27846	0.08688	0.38207
Minimum [mm]	21.6832	4.12312	7.66351
Maximum [mm]	21.9616	4.21	8.04558
Count	30	30	30
Coefficient of variation [%]	0.391%	0.766%	1.292%

**Table 25:** Statistical data based on measurements for a 10 kg load in the five-axis configuration in Position III.

	NCD 50 A	NCD 50 B	NCD 10
Mean [mm]	13.5129	10.1821	2.0524
Standard error [mm]	0.01223	0.00447	0.01401
Median [mm]	13.5059	10.1852	2.06785
First quartile [mm]	13.4623	10.163	2.00576
Third quartile [mm]	13.5727	10.2011	2.10238
Variance [mm^2^]	0.00449	0.0006	0.00589
Standard deviation [mm]	0.067	0.0245	0.07672
Kurtosis	−0.92241	−0.3202	0.11071
Skewness	0.02243	−0.27433	−0.7236
Range [mm]	0.24929	0.10267	0.29531
Minimum [mm]	13.3939	10.1241	1.86148
Maximum [mm]	13.6432	10.2268	2.15678
Count	30	30	30
Coefficient of variation [%]	0.496%	0.241%	3.738%

**Table 26:** Statistical data based on measurements for a 10 kg load in the five-axis configuration in Position IV.

	NCD 50 A	NCD 50 B	NCD 10
Mean [mm]	23.9531	32.0037	2.89637
Standard error [mm]	0.00689	0.00286	0.01495
Median [mm]	23.9575	32.0038	2.90767
First quartile [mm]	23.9271	31.9945	2.84038
Third quartile [mm]	23.982	32.0186	2.96462
Variance [mm^2^]	0.00142	0.00025	0.00671
Standard deviation [mm]	0.03771	0.01566	0.08189
Kurtosis	−0.31038	−0.41216	−0.82637
Skewness	0.20653	−0.51366	−0.52583
Range [mm]	0.15281	0.05535	0.28227
Minimum [mm]	23.8922	31.9712	2.73734
Maximum [mm]	24.045	32.0266	3.01962
Count	30	30	30
Coefficient of variation [%]	0.157%	0.049%	2.827%

**Table 27:** Statistical data based on measurements for a 6 kg load in the six-axis configuration in Position I.

	NCD 50 A	NCD 50 B	NCD 10
Mean [mm]	33.518762	24.7965	5.22126
Standard error [mm]	0.0092384	0.00925	0.01325
Median [mm]	33.512323	24.779	5.22313
First quartile [mm]	33.477904	24.7624	5.17245
Third quartile [mm]	33.562722	24.8337	5.25144
Variance [mm^2^]	0.0025604	0.00257	0.00527
Standard deviation [mm]	0.0506007	0.05067	0.07257
Kurtosis	−1.215567	−0.67197	1.92114
Skewness	0.263029	0.44423	0.60616
Range [mm]	0.1633671	0.18678	0.36299
Minimum [mm]	33.445585	24.7203	5.08031
Maximum [mm]	33.608952	24.9071	5.4433
Count	30	30	30
Coefficient of variation [%]	0.151%	0.204%	1.390%

**Table 28:** Statistical data based on measurements for a 6 kg load in the six-axis configuration in Position I.

	NCD 50 A	NCD 50 B	NCD 10
Mean [mm]	16.6546	23.8457	3.52075
Standard error [mm]	0.01566	0.01123	0.01861
Median [mm]	16.6551	23.8338	3.48511
First quartile [mm]	16.5895	23.7873	3.42777
Third quartile [mm]	16.72	23.9088	3.61178
Variance [mm^2^]	0.00735	0.00378	0.01039
Standard deviation [mm]	0.08576	0.06149	0.10192
Kurtosis	−0.80961	−1.51619	−1.40078
Skewness	−0.31733	0.37391	0.20228
Range [mm]	0.3087	0.17103	0.32899
Minimum [mm]	16.4689	23.7755	3.38789
Maximum [mm]	16.7776	23.9465	3.71688
Count	30	30	30
Coefficient of variation [%]	0.515%	0.258%	2.895%

**Table 29:** Statistical data based on measurements for a 6 kg load in the six-axis configuration in Position III.

	NCD 50 A	NCD 50 B	NCD 10
Mean [mm]	28.897	12.8214	0.90873
Standard error [mm]	0.02659	0.00589	0.0282
Median [mm]	28.9164	12.8177	0.88995
First quartile [mm]	28.7704	12.7949	0.82727
Third quartile [mm]	29.026	12.8519	0.94218
Variance [mm^2^]	0.0212	0.00104	0.02386
Standard deviation [mm]	0.14561	0.03228	0.15447
Kurtosis	−1.22059	−1.12565	7.44615
Skewness	−0.13247	−0.06511	2.21585
Range [mm]	0.49502	0.10933	0.80914
Minimum [mm]	28.6709	12.7628	0.70363
Maximum [mm]	29.1659	12.8721	1.51277
Count	30	30	30
Coefficient of variation [%]	0.504%	0.252%	16.998%

**Table 30:** Statistical data based on measurements for a 6 kg load in the six-axis configuration in Position III.

	NCD 50 A	NCD 50 B	NCD 10
Mean [mm]	19.8174	3.09805	3.53404
Standard error [mm]	0.0109	0.00209	0.01758
Median [mm]	19.8281	3.09809	3.54761
First quartile [mm]	19.7737	3.08949	3.4707
Third quartile [mm]	19.8589	3.10579	3.61222
Variance [mm^2^]	0.00356	0.00013	0.00927
Standard deviation [mm]	0.0597	0.01143	0.09629
Kurtosis	0.23433	−0.6626	−0.67573
Skewness	−0.67939	0.35859	−0.53119
Range [mm]	0.24505	0.04336	0.33813
Minimum [mm]	19.6621	3.07957	3.35185
Maximum [mm]	19.9072	3.12292	3.68998
Count	30	30	30
Coefficient of variation [%]	0.301%	0.369%	2.725%

## Data Availability

The original contributions presented in this study are included in the article. Further inquiries can be directed to the corresponding author.
